# Determination of vat-photopolymerization parameters for microneedles fabrication and characterization of HPMC/PVP K90 dissolving microneedles utilizing 3D-printed mold

**DOI:** 10.1038/s41598-024-67243-y

**Published:** 2024-07-13

**Authors:** Baramee Chanabodeechalermrung, Tanpong Chaiwarit, Suruk Udomsom, Pornchai Rachtanapun, Promporn Piboon, Pensak Jantrawut

**Affiliations:** 1 Department of Pharmaceutical Sciences, Faculty of Pharmacy, Chiang Mai University, Chiang Mai, 50200 Thailand; 2 Biomedical Engineering and Innovation Research Center, Chiang Mai University, Chiang Mai, 50200 Thailand; 3 Biomedical Engineering Institute (BMEI), Chiang Mai University, Chiang Mai, 50200 Thailand; 4 Office of Research Administration, Chiang Mai University, Chiang Mai, 50200 Thailand; 5 Division of Packaging Technology, School of Agro-Industry, Faculty of Agro-Industry, Chiang Mai University, Chiang Mai, 50100 Thailand; 6 Center of Excellence in Materials Science and Technology, Chiang Mai University, Chiang Mai, 50200 Thailand; 7 Faculty of Veterinary Medicine, Chiang Mai University, Chiang Mai, 50100 Thailand; 8 Center of Excellence in Agro Bio-Circular-Green Industry (Agro BCG), Agro-Industry, Chiang Mai University, Chiang Mai, 50100 Thailand

**Keywords:** Drug delivery, Techniques and instrumentation, Structural materials

## Abstract

Three-dimensional (3D) printing serves as an alternative method for fabricating microneedle (MN) patches with a high object resolution. In this investigation, four distinct needle shapes: pyramid mounted over a long cube (shape A), cone mounted over a cylinder (shape B), pyramidal shape (shape C), and conical shape (shape D) were designed using computer-aided design (CAD) software with compensated bases of 350, 450 and 550 µm. Polylactic acid (PLA) biophotopolymer resin from eSun and stereolithography (SLA) 3D printer from Anycubic technology were used to print MN patches. The 3D-printed MN patches were employed to construct MN molds, and those molds were used to produce hydroxypropyl methylcellulose (HPMC) and polyvinyl pyrrolidone (PVP) K90 dissolving microneedles (DMNs). Various printing parameters, such as curing time, printing angle, and anti-aliasing (AA), were varied to evaluate suitable printing conditions for each shape. Furthermore, physical appearance, mechanical property, and skin insertion ability of HPMC/PVP K90 DMNs were examined. The results showed that for shape A and C, the suitable curing time and printing angle were 1.5 s and 30° while for shapes B and D, they were 2.0 s and 45°, respectively. All four shapes required AA to eliminate their stair-stepped edges. Additionally, it was demonstrated that all twelve designs of 3D-printed MN patches could be employed for fabricating MN molds. HPMC/PVP K90 DMNs with the needles of shape A and B exhibited better physicochemical properties compared to those of shape C and D. Particularly, both sample 9 and 10 displayed sharp needle without bent tips, coupled with minimal height reduction (< 10%) and a high percentage of blue dots (approximately 100%). As a result, 3D printing can be utilized to custom construct 3D-printed MN patches for producing MN molds, and HPMC/PVP K90 DMNs manufactured by those molds showed excellent physicochemical properties.

## Introduction

Three-dimensional (3D) printing is an additive manufacturing (AM) technology printing the object layer by layer. Computer-aided design (CAD) software is initially utilized to design the 3D model following by converting the 3D model to the standard triangulation language (STL) format, which is the standard and most frequently used format^1^. The designed model is then sliced into layers^2^ before sending to a 3D printer, and each sliced layer is constructed by a 3D printer^1,3^ initiating from the base layer and constructing a series of layers on top^2^. 3D printing plays an important role in object fabrication with a high complexity in a fast and efficient method^4^. Importantly, 3D printing can be used to construct microneedle (MN) patches instead of the conventional method which is micro-electromechanical systems (MEMS). Although MEMS is an effective technology for micro-device fabrication because this technology has the potential for mass production of those micro-devices, it has complex muti-step of production, and requires specialize training, including high cost of the machine^5^. Thus, 3D printing can be utilized to fabricate 3D objects rather than MEMS since it can construct a high-resolution object in a fast production, including easy to scale up and unnecessary special training need^3,5^.

In vat-photopolymerization, a light-curable polymer resin is treated with either visible light or ultraviolet (UV) light initiating polymerization reaction, which the polymer chains are formed into a solid resin, which is irreversible and cannot be changed back to the liquid form^1^. Noteworthy, utilization of vat-photopolymerization techniques, such as stereolithography (SLA) and digital light processing (DLP)^2^ has been reported for printing MN patch^3^ because of a low cost of 3D printer and these two technologies give high resolution 3D objects. In DLP, digital light projector is used instead of a mirror, which is employed in SLA, to scatter a laser source accelerating the printing process^1^. Due to the same resolution of the object, utilization of SLA printers tends to be more affordable than DLP printers since SLA printers are cheaper than DLP printers^3^. At present, directly 3D-printed MN via vat photopolymerization shows some drawbacks, such as photo-absorber toxicity, and unable to incorporate drug during printing process^6^. Moreover, vat photopolymerization can only produce solid MN since the polymer resin solidifies after contacting light, and it cannot change the shape structure after completely solidifying^1^. However, SLA can be used to construct solid MN with the desired height, diameter and shape, and those of 3D-printed solid MNs can be utilized to fabricate MN mold. Nowadays, vat photopolymerization had been reported for printing pyramidal and cone shape microneedles^7–10^ by adjusting printing angle^7,9^, needle height^7^ and needle to needle spacing^9,11^. However, there are three essential parameters, such as curing time^12^, printing angle^9,13^ and anti-aliasing^14^ that impacted needles appearance because these three vital parameters influence the size^12,15^, sharpness^13^, shape and smoothness of the needles^16^.

Microneedle (MN) is described as a small device used for transdermal drug delivery system^17^, and normally ranged from 25 to 2000 μm in height which are attached to a base support^5,18^. Utilization of MN patch for creating small holes on the skin can be used to deliver macromolecules or possibly supramolecular complexes^5^, which cannot cross skin barrier by topical application. On the other word, drug-loaded MN can temporarily penetrate the stratum corneum improving skin permeability and subsequently enhance penetration efficiency of the drug for greater therapeutic effect^17,19^ since the utilization of MN to deliver drugs can avoid first-pass metabolism occurring at liver^20^. Interestingly, MNs are well-known as a painless device because they penetrate through the epidermis layer but not deep into the dermis layer where the nociceptive fibers and blood vessels located^3,20^. However, MN can be utilized to deliver drugs or substances into the systemic circulation by directly leading the drugs to the upper dermis layer, so the drugs can directly jump into the systemic circulation without facing the skin barrier^21^. Determined by delivery approaches, MNs are categorized into five types, namely solid MNs, hollow MNs, coated MNs, dissolving MNs, and hydrogel-forming MNs^18^.

Dissolving microneedles (DMNs) is used to deliver water-soluble therapeutic drugs into the skin with the “poke and dissolve” approach. DMNs are usually produced from biocompatible, biodegradable, water soluble and low-cost polymers^22^, such as sucrose, hyaluronic acid, polyvinyl pyrrolidone (PVP) and hydroxypropyl methylcellulose (HPMC)^3,23^. DMNs should dissolve after contact with biological fluid including water, and subsequently release the drugs dispersed in the needles^24^ through the skin, without the remaining shape of DMNs left behind^23^. Moreover, DMNs do not need to be removed after insertion so they can increase patient compliance. However, complete insertion and dissolution of DMNs are one of the challenging part because the dissolution rate and DMNs strength depending on the type of polymers^8^. Currently, DMNs are primarily manufactured using molding method which was considered as one of the most suitable method for constructing DMNs^8^ because molding method is cheap, easy to scale up and incorporate drug^25^, reproducible^26^ and no special training require^25,26^. However, customizability and design complexity of microneedle are the limitation of molding method^26^. Nowadays, 3D printing especially vat-photopolymerization cannot be used to print DMNs because most of the polymer resins available in the market were not formulated with biodegradable polymers^26^. Additionally, most of the photo-absorbers utilized in vat-photopolymerization are toxic^6^.

In this research, 3D printing technique (vat photopolymerization) was used to produce a 3D-printed MN patch with four different shapes of needle. Furthermore, the three important parameters that impact the needle appearance; curing time, printing angle and anti-aliasing were evaluated to ensure that the 3D-printed MN patches matched the designed model from AUTODESK^®^ FUSION 360™ software. Moreover, the three printing parameters utilized to print the four different shapes of MN reported in this study can be used to construct MN in the desired shape, diameter and height. Subsequently, 3D-printed MN patches were utilized to construct MN molds, and these MN molds were used to manufacture DMNs patch using hydroxypropyl methylcellulose (HPMC) and polyvinyl pyrrolidone (PVP) K90 solution. Lastly, some important factors of DMNs, such as physical appearance, %height reduction and skin insertion study were evaluated.

## Methods

### Ethics information

This study is reported in accordance with ARRIVE guidelines and was approved to utilize the neonatal porcine skin obtained from natural death porcine in a local farm in Lamphun province by Faculty of Veterinary Medicine Chiang Mai University ANIMAL CARE AND USE COMMITTEE (FVM-ACUC): Ref. No. R24/2566, from February 1, 2024, to December 31, 2024.

### Materials

Acrylic-based resin (eResin PLA biophotopolymer resin) was purchased from eSun Industrial Co., Ltd., Shenzhen, China). Polydimethylsiloxane (PDMS) was obtained from Dow Deutschland Inc., Berlin, Germany. Hydroxypropyl methylcellulose (HPMC) E50 was received from Onimax Co., Ltd., Bangkok, Thailand. Polyvinylpyrrolidone (PVP) K90 and isopropyl alcohol (IPA) were acquired from Union Science Co., Ltd., Chiang Mai, Thailand. Ethanol was purchased from RCI lab scan, Ltd., Bangkok, Thailand. Deionized water (DI) served as a solvent to prepare MN patches.

### The design of MN patch

**Figure 1:**
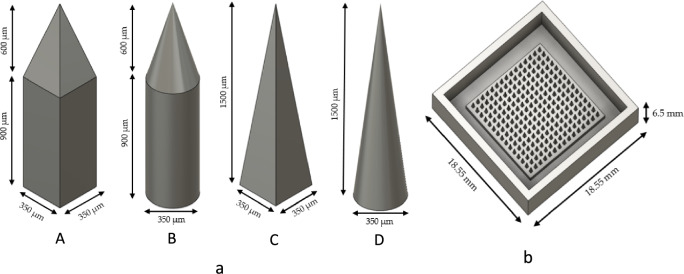
Design of four different shapes of needle (**a**) and MN patch (**b**) using AUTODESK^®^ FUSION 360TM Software version 2.0.16985.

The needle shape on the MN patch was designed using AUTODESK^®^ FUSION 360TM Software version 2.0.16985 (Autodesk Incorporation, San Rafael, CA, USA) into four distinct shapes: pyramid mounted over a long cube (shape A), cone mounted over a cylinder (shape B), pyramidal shape (shape C), and conical shape (shape D) with the height of 1500 μm and a diameter or width of 350 μm, as depicted in Fig. 1a. The designed MNs patch comprised an array of 15 × 15 needles with a 450 μm spacing between the needles as illustrated in Fig. 1b. Following the determination of suitable printing parameters for each design, the four different needle shapes were adjusted to have a diameter or width of 450 and 550 μm with the same height (1500 μm). Subsequently, the designed MNs patches were printed using the suitable printing parameters for each shape.

### Determination of 3D printing parameter and physical characterization of 3D printed MNs patch

After MNs patches were designed, the computer-aided design (CAD) files of the MNs patches were converted into STL files before being sent to the Slicer Program, which is used to determine interested printing parameters, and then sent to the stereolithography (SLA) 3D printer (Anycubic Photon Mono X 6Ks, Shenzhen Anycubic Technology Co., Ltd., Shenzhen, China) to print MNs patch for evaluating the suitable parameters. Three interested parameters were utilized in printing process: curing time of printing, printing angle, and anti-aliasing because these parameters impacted size, sharpness, shape and smoothness of the needles. Firstly, the designed MNs patches were printed using the curing times of 1.0, 1.5, and 2.0 s in each design without changing the angle of the model on the build plate (0°). Secondly, after determining suitable curing time for each design, the MNs patch was printed using three different angles: 30°, 45°, and 60°, with and without the anti-aliasing. The MNs patch was printed using acrylic-based resin (eResin PLA biophotopolymer resin, eSun, Shenzhen, China). Upon completion of the printing process, the MNs patches were rinsed with isopropyl alcohol (IPA) to eradicate residual resin and then cured using the ANYCUBIC Cure Machine 2.0 ultraviolet (UV) light-emitting diode (LED) lamp (Anycubic Technology, Hong Kong, China) for 20 min to complete cure and solidify the MNs patch. Subsequently, the 3D-printed MNs patch was photographed utilizing an inverted microscope (BDS400-FL2, Chongqing Drawell Instrument co. Ltd., Chongqing, China), and ImageJ program was employed for measuring the width/diameter and height of the needles.

### 3D printing of MNs patch

Four different shapes of MN at the width/diameter of 350, 450 and 550 µm were constructed using the suitable printing parameters for each shape using acrylic-based resin (eResin PLA biophotopolymer resin, eSun, Shenzhen, China) employing the methods in "Determination of 3D printing parameter and physical characterization of 3D printed MNs patch". At the width/diameter of 350 µm, shape A, B, C and D were given the code name as Design 1, 2, 3 and 4, respectively. At the width/diameter of 450 µm, shape A, B, C and D were given the code name as Design 5, 6, 7 and 8, respectively. Lastly, at the width/diameter of 550 µm, shape A, B, C and D were given the code name as Design 9, 10, 11 and 12, respectively.

### Fabrication of reverse PDMS MNs molds

After obtaining the 3D-printed MNs patches, all 12 designs were utilized to fabricate PDMS molds using a liquid-phase PDMS mixed with curing agent at a ratio of 10:1. Subsequently, the liquid-phase PDMS was poured onto the 3D-printed MNs patches, and a vacuum pump (Trivac D16T, Leybold Dresden GmbH, Dresden, Germany) was employed to remove air bubbles from the liquid-phase PDMS. A hot air oven (Model ED 23, Binder, Tuttlingen, Germany) was utilized to cure the PDMS at 60 °C for 4 h. Finally, an inverse PDMS mold was carefully removed from the 3D-printed MNs patch.

### Preparation of HPMC/PVP K90 solution and fabrication of MNs patches

After the fabrication of molds, MN patches were prepared utilizing two water-soluble polymers, namely hydroxypropyl methylcellulose (HPMC) E50 and polyvinylpyrrolidone (PVP) K90 which were selected from the previous study^3^. Initially, a solution of 7% w/w HPMC E50 and a solution of 40% w/w PVP K90 were individually dissolved in a solvent comprising ethanol and water in a ratio of 8:2. This solvent composition was chosen to expedite the drying process of the DMNs patches. Subsequently, the HPMC E50 solution and the PVP K90 solution were combined at a ratio of 1:2. The molds along with the polymer solution were then placed into a centrifuge tube, and the solution was forced into the small cavities of the molds using a centrifuge machine (MPW-352R, Warsaw, Poland) operating at 6000 rpm for a duration of 1 h. Following centrifugation, the MN molds were removed from the centrifuge tube and left at ambient temperature for 24 h. Finally, the MN patches were carefully extracted from the molds. At the mold cavities width/diameter of 350 µm, shape A, B, C and D were given the sample name as Design 1, 2, 3 and 4, respectively. At mold cavities width/diameter of 450 µm, shape A, B, C and D were given the sample name as Design 5, 6, 7 and 8, respectively. Lastly, at the mold cavities width/diameter of 550 µm, shape A, B, C and D were given the sample name as Design 9, 10, 11 and 12, respectively.

### Physical characterizations

#### Morphological characterization

The examination of the needle structure was conducted employing a scanning electron microscope (JEOL JCM-7000 NeoScopeTM Benchtop, Tokyo, Japan) operating at 15 kV under high vacuum condition. To facilitate observation, the MN patch was cut and attached to aluminum stub utilizing a double-sided adhesive carbon tape. Subsequently, all MN patches were coated with gold for 1 min prior to the SEM operation at a magnification of 45 × both top and front view.

#### Percentage of height reduction

Height reduction (%) of the needles was performed using a Texture Analyzer, TA.XT plusC (Stable Micro Systems, Surrey, UK). The needles were taken a photo using a RS PRO USB digital microscope (RS PRO, Bangkok, Thailand) and measured the height of the needle before and after a compression mode using ImageJ software version 1.8.0. The MN patch was tied to a stainless-steel platform using a double-sided adhesive tape, and the probe was compressed at a speed of 0.1 mm/s to the stainless-steel platform. Moreover, the pre- and post-test speeds were predetermined at 1 and 10 mm/s, respectively. Lastly, a high reduction (%) was calculated using the following Eq. (1) below.

1 $$\text{Height reduction }(\text{\%}) = \frac{\text{Height before compression}-\text{Height after compression}}{\text{Height before compression}}\times 100$$

#### Skin insertion study

Neonatal porcine skin and phosphate buffer saline pH 7.4 were utilized as a human skin and biological fluid, respectively to determine skin insertion ability of DMNs patch. Neonatal porcine skin was obtained from natural death piglets in a local farm in Lamphun, Thailand. Moreover, this study followed the ethical guidelines, and was approved by Faculty of Veterinary Medicine Chiang Mai University ANIMAL CARE AND USE COMMITTEE (FVM-ACUC): Ref. No. R24/2566. Firstly, tissue paper saturated with PBS pH 7.4 was placed on PDMS block, and then covered with neonatal porcine skin. Secondly, MN patch was pressed on the skin using thumb press for 30 s. Thirdly, 0.1%w/v methylene blue was applied on the skin for 10 min, and then washed using PBS pH 7.4 to remove the residual methylene blue. Finally, the number of blue dots that appeared on the skin were counted and the blue dot percentage (% blue dots) was calculated using Eq. (2) below.

2 $$\text{\% Blue dots }= \frac{\text{The number of blue dots appeared on the skin}}{\text{The number of needles of MN}} \times 100$$

### Statistical analysis

The results were reported as average value ± standard deviations (S.D.) using SPSS software version 17.0 (IBM Corporation, Armonk, NY, USA) for statistical analysis. Moreover, a one-way ANOVA test was employed to evaluate the disparity among the datasets. Statistically significant difference was determined when the *p*-value is less than 0.05.

## Results and discussion

### 3D printing parameter determination and physical characterization of 3D-printed MNs patch

Initially, the designed MNs patches with four distinct shapes denoted as shapes A, B, C, and D with width/diameter of 350 μm (design 1 to design 4) were used to assess the optimal printing conditions for each shape. After acquiring 3D-printed MNs patches under varying printing parameter, the 3D-printed MNs patches were measured their height and width/diameter using an inverted microscope. This part of study focused on the evaluation of the appropriate printing parameters, which are curing time, printing angle and anti-aliasing.

#### Curing time determination

**Figure 2:**
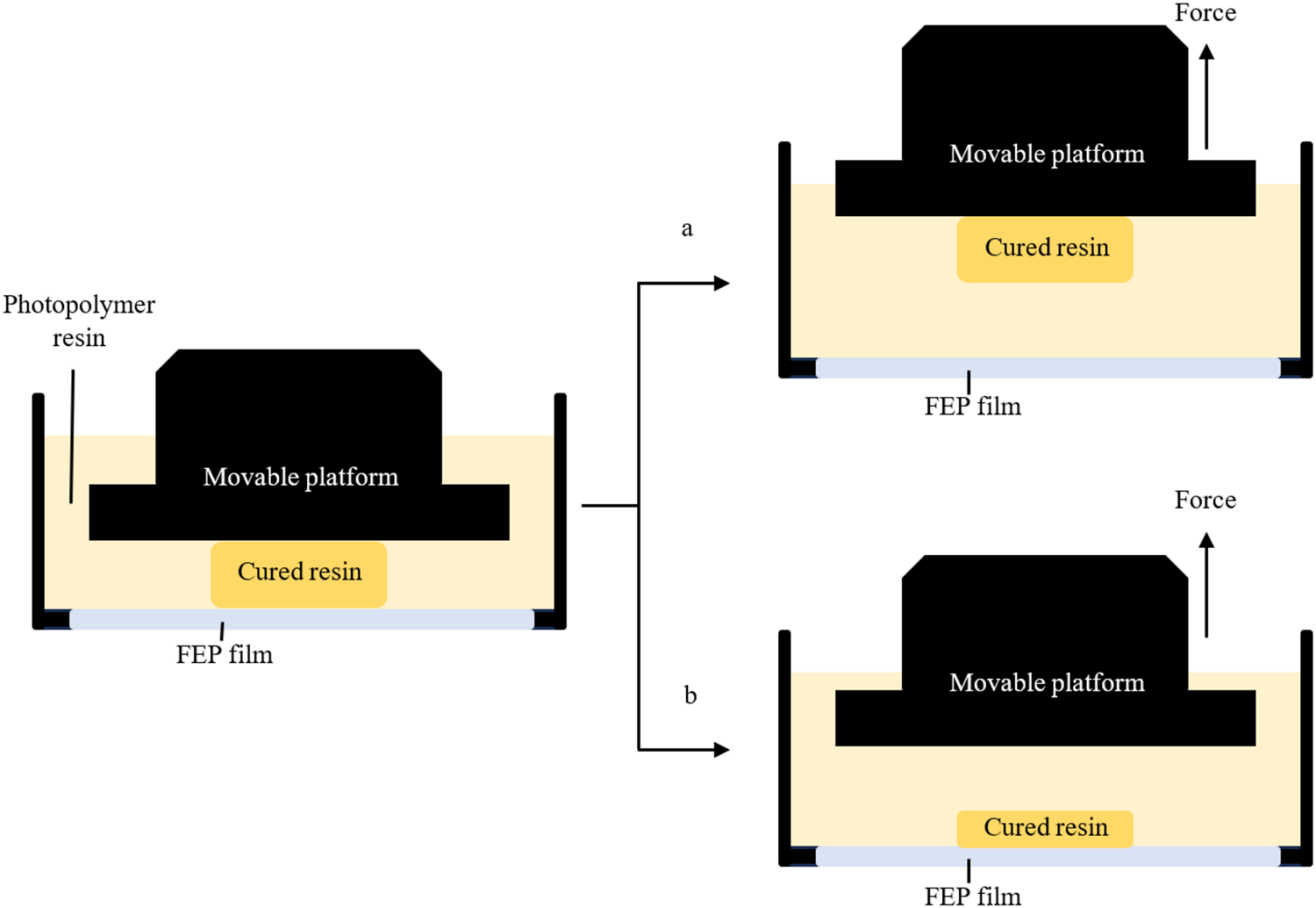
Scheme diagram of interface separation because of the platform moving upward in bottom-up vat-photopolymerization. (**a**) normal printing process. (**b**) Printing failure.

Curing time, a critical parameter, denotes the temporal requisite duration for UV light to initiate polymerization within a liquid resin^15^. Moreover, curing time impacts size of the needles since the prolonged curing time influences the higher amount of liquid resin contacted to the UV light^27^ resulting in larger needle structure influencing high tensile strength of the object^12^. During the polymerization phrase, the cured resin robustly attached to both the transparent fluorinated ethylene propylene (FEP) film and movable platform, and when the separation force was applied, the cured resin was separated from FEP film (Fig. 2a). In this research, the impact of different curing time on the fabrication process was evaluated, and the designed MNs patches were printed at 0° angle using three different curing times: 1.0, 1.5 and 2.0 s. Interestingly, the results showed that a curing time of 1.0 s was insufficient for curing the 3D-printed MNs patches because all shapes of the needle (shape A, B, C and D) were not able to print. Because of the low curing time, the polymer resin cannot be cured properly^28^ resulting in low adhesion to the movable platform^12^. Consequently, the cured resin strongly attached to the FEP film instead of the movable platform (Fig. 2b) leading to printing failures. To ensure strong adhesion to the movable platform and previous cured layers, a longer curing time is necessary to overcome the separation force^28^. However, an excessive curing time can damage the surface of the 3D-printed object and makes the 3D-printed model inaccurate^15,28^.

It is notable that at a curing time of 1.5 s, only shape A and C were successfully fabricated while those with round base (shape B and D) failed to print. Shape A and C had a width base at 327.68 ± 14.11 and 328.08 ± 13.33 μm, respectively as illustrated in Table 1. Obviously, the square base possesses a larger surface area than the round base in the same diameter or width so the utilization volume of polymer resin to print square base MN is higher than round base MN affecting the higher amount of monomer participated in polymerization reaction. Importantly, if the amount of monomer participated in polymerization reaction is inadequate, the oxygen can effectively diffuse through the polymer resin resulting in termination of polymerization^15^. Hence, the decrease in the volume of pre-polymer solution can be solved by extending the duration of UV light exposure to facilitate a more robust polymerization process^15,28^.

**Table 1:** Base width/diameter of design A, B, C and D at the curing time of 1.5 and 2.0 s.

Curing time (s)	Base width/diameter (μm)			
	Shape A	Shape B	Shape C	Shape D
1.5	327.68 ± 14.11^a^	–	328.08 ± 13.33^a^	–
2.0	401.47 ± 16.87^b^	331.61 ± 16.47^a^	394.99 ± 21.10^b^	332.05 ± 16.44^a^

For each test, the average values with the same letter show no statistical difference (*p* > 0.05).

For a curing time of 2.0 s, all needle shapes were capable of printing. However, the base width of shapes A and C was 401.47 ± 16.87 and 394.99 ± 21.10 μm, respectively, whereas for shapes B and D, the diameter was 331.61 ± 16.47 and 332.05 ± 16.44 μm, respectively as shown in Table 1. Consequently, the optimal curing time for shapes A and C was determined to be 1.5 s, while for shapes B and D, it was determined to be 2.0 s. It can be inferred that an increase in curing time led to the enlargement of the needle size, a finding consistent with the results reported by Nurlatifah et al.^27^. However, it is noteworthy that each shape exhibited an unacceptable physical appearance since all shapes showed stair-stepped edges as illustrated in Table 2. Moreover, the height of the needle was not the same as the design from AUTODESK^®^ FUSION 360™ Software.

**Table 2:** 3D-printed MN structure at the angle of 0°, using 1.5 and 2.0 s of curing time photographed by an inverted microscope at 10 × magnification.

Curing time (s)	Shape A	Shape B	Shape C	Shape D
1.5		–		–
2.0				

#### Printing angle determination

**Figure 3:**
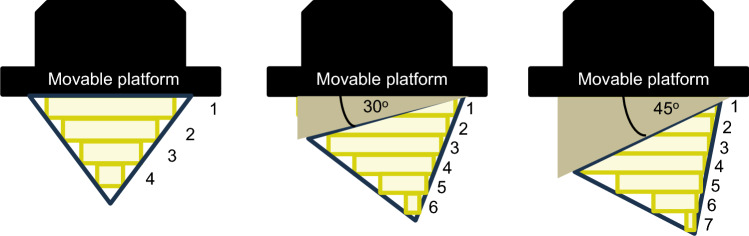
Concept of printing angle on the number of printing layers and needle sharpness.

Following the determination of appropriate curing time for shapes A, B, C, and D, MN patches were printed utilizing different printing angles: 30°, 45° and 60°. The printing angle affected the height and width/diameter of the needles including sharpness owing to the distinct layers observed at each angle^9^ as illustrated in Fig. 3. On the other word, higher printing angle had a greater number of printing layers compared to lower printing angle. Noteworthy, the latest printing layer, particularly a smaller layer, may not strongly attach to the previous cured layers because of the reduction of polymer resin volume involved in the polymerization reaction. Hence, the polymer resin was not cured properly, and firmly attached to FEP film instead of the previous cured layers^15^.

After shape A and C were printed at a curing time of 1.5 s, the results showed that an optimal printing angle was 30° since the base width of shape A and C was 343.11 and 345.61 μm, respectively, which was close to 350 μm as same as the design from AUTODESK^®^ FUSION 360™ Software. Conversely, shape B and D were printed with a curing time of 2.0 s, and the results showed that the optimal printing angle was 45° because the diameter of shape B and D was 346.04 and 348.98 μm (Table 3), respectively, which was approximately 350 μm as same as the design. At 60°, all shapes had the base width/diameter more than 350 μm, which was more than the design. This phenomenon can be attributed to the larger surface area contact between the previous layers at higher angle resulting in a greater deposition of material leading to a wider base/diameter of the 3D-printed MN structure^15^. Thus, it can be concluded that printing angle affected the base width/diameter of the 3D-printed MN structure. Specifically, higher printing angle was associated with increased base width/diameter as demonstrated in Table 3. Notably, the suitable printing angle for shapes A and C was 30°, while for shapes B and D, it was 45°. The results found that the different shapes of the needles required different printing angle to produce MNs patch. The results had been influenced by the base shape of MN because shape A and C were a square base, while base shape B and D were a round base. Furthermore, the square-based needle had a larger printing volume compared to the round-based needle, so the square-based needle required less material deposition than the round-based needle. Moreover, a high material deposition can be obtained by adjust the printing angle^9,15^. Nevertheless, the edges quality of the four different shapes of needle was not acceptable since all designs showed stair-stepped edges as demonstrated in Table 4.

**Table 3:** Base width/diameter of shape A, B, C and D using 30°, 45° and 60° of printing angle.

Printing angle (°)	Base width/diameter (μm)			
	Shape A	Shape B	Shape C	Shape D
30	343.11 ± 9.53^a^	338.95 ± 10.39^a^	345.61 ± 11.52^a^	333.75 ± 12.44^a^
45	367.33 ± 13.46^b^	346.04 ± 11.26^a^	365.06 ± 12.23^a,b^	348.98 ± 9.57^a^
60	380.47 ± 10.25^b^	376.90 ± 12.60^b^	381.16 ± 11.96^b^	373.34 ± 9.33^b^

For each test, the average values with the same letter show no statistical difference (*p* > 0.05).

**Table 4:** Four distinct shapes of 3D-printed MN at the printing angle of 30°, 45° and 60° under an inverted microscope at 10 × magnification.

Printing angle	Shape A	Shape B	Shape C	Shape D
30°				
45°				
60°				

#### Anti-aliasing determination

After acquiring appropriate curing time and printing angle, the designed MNs patches were printed with anti-aliasing aimed to eradicate stair-stepping edges. Interestingly, the objects printed via vat-photopolymerization technique often exhibited surface imperfection, such as stair-stepping edges because of the layer-based construction process, and the alias curing images produced from pixel-based laser systems^16^. The results showed that shape A, B, C and D printed with anti-aliasing revealed a width/diameter base of 345.47, 345.19, 341.68 and 339.78 µm, respectively as illustrated in Table 5. Moreover, all four shapes illustrated smooth edges without broken structure or bent tips as shown in Fig. 5. However, the height of shape A, B, C and D was 1426.45, 1421.98, 1395.71 and 1389.52 μm, respectively (Table 5), which was not matched to the designs (1500 μm) from CAD software since the higher layers were very tiny so they were not cured properly resulting in attaching to FEP film instead of the previous cured layers^28^. Although the needle height did not match the design, if the needles are sharp and capable of piercing the skin to deliver the drugs, the height discrepancy is not a problem. Thus, all twelve 3D-printed MN patches can be used to construct MNs molds.

**Table 5:** Base width/diameter (μm) and height (μm) of twelve designs.

Design	Base width/diameter (μm)	Relative error (%)	Height (μm)	Relative error (%)
1	345.47	1.30	1426.45	4.90
2	345.19	1.37	1421.98	5.20
3	341.68	2.38	1395.71	6.95
4	339.78	2.92	1389.52	7.37
5	447.82	0.49	1449.71	3.35
6	440.50	2.11	1440.09	3.99
7	441.02	1.99	1414.81	5.68
8	440.47	2.12	1406.77	6.22
9	548.87	0.21	1482.62	1.16
10	543.27	1.22	1472.72	1.82
11	546.49	0.64	1452.66	3.16
12	540.29	1.77	1441.33	3.91

**Figure 4:**
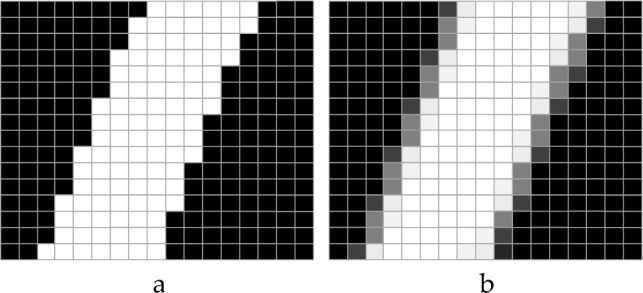
Concept of anti-aliasing shown by the line without (**a**) and with (**b**) anti-aliasing.

Anti-aliasing (AA) is an essential technique related to imaging aimed for improving spatial resolution^14^ by removing the occurrence of jagged, or stair-stepped lines along object edges and making the smooth object edges. The reduction of stair-stepped edges relates to the light from each pixel that propagates to the polymer resin leading to the enlarged and rounded pixels. Due to the convolution of the focused image, each pixel blurs with surrounding pixels influencing smooth and continuous pattern^29^ as illustrated in Fig. 4. Thus, the application of anti-aliasing can reduce stair-stepping edges on MNs surface that bears significant concern about mechanical strength of MNs.

In conclusion, the suitable printing parameters for shape A and C were 1.5 s of curing time, 30° printing angle and anti-aliasing whereas for shape B and D, 2.0 s of curing time, 45° printing angle and anti-aliasing were the appropriate printing parameters.

### 3D printing of MNs patch

**Figure 5:**
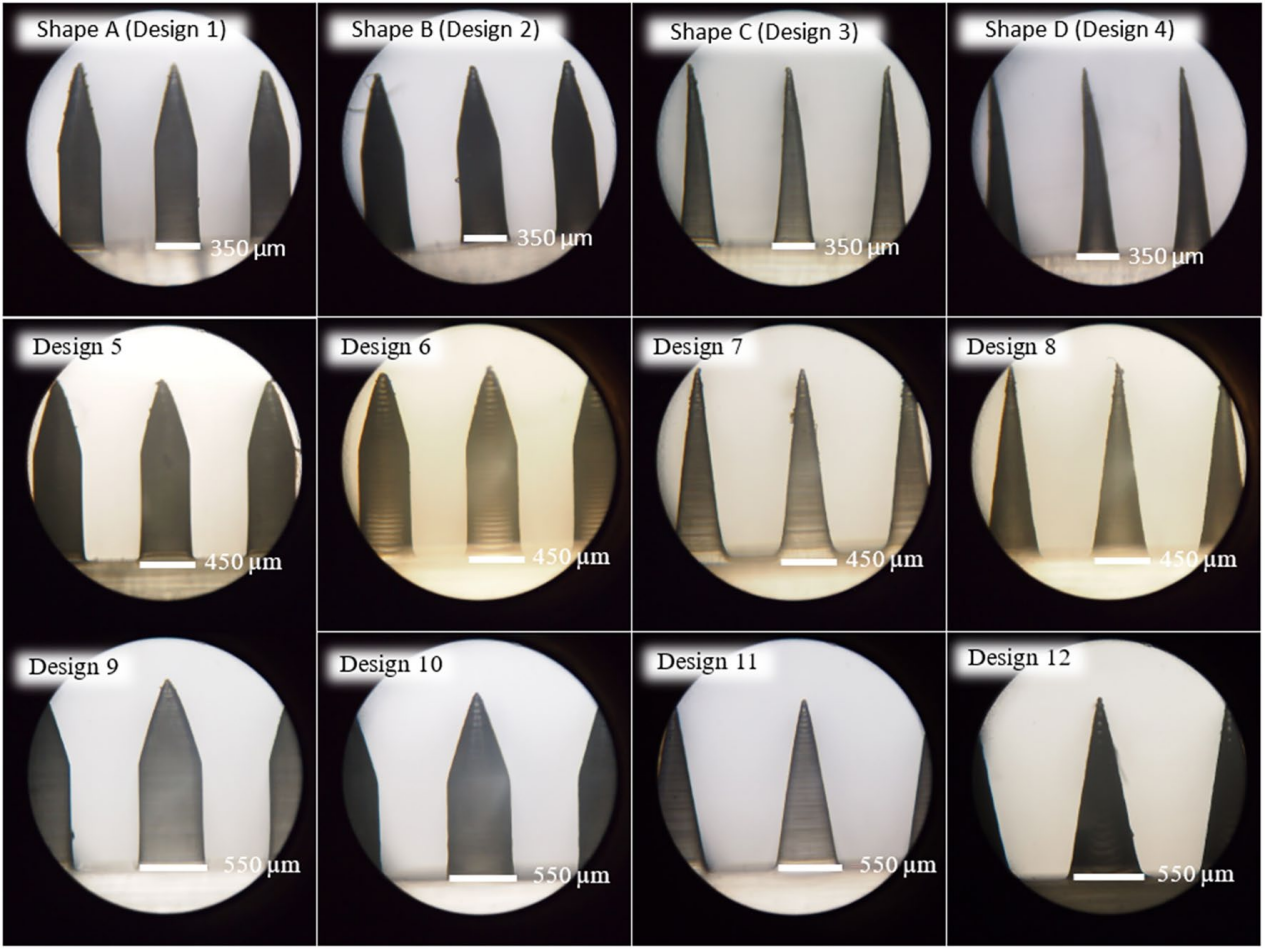
The physical appearance of twelve designs photographed by an inverted microscope at 10 × magnification.

After obtaining suitable printing parameters for each needle shape, designs 1, 3, 5, 7, 9, and 11 were constructed using 1.5 s of curing time, 30° printing angle, and employing anti-aliasing technique. Conversely, designs 2, 4, 6, 8, 10, and 12 were printed with a curing time of 2.0 s, a printing angle of 45° and anti-aliasing. The outcomes illustrated that all twelve designs were successfully fabricated 3D-printed MNs patches with an acceptable appearance, such as sharp needle, neither broken structure nor bend tip as depicted in Fig. 5. Nevertheless, the results illustrated that all twelve 3D-printed MN patches exhibited needle height less than 1500 μm but exceed 1400 μm as illustrated in Table 5. However, an increase in width/diameter of the designs can increase the needle height because the higher layers of large base width/diameter of needles are bigger than those of small base width/diameter^30^. In conclusion, all 3D-printed MN patches can be used to fabricate MN molds.

### Fabrication of reverse PDMS MNs molds

**Figure 6:**
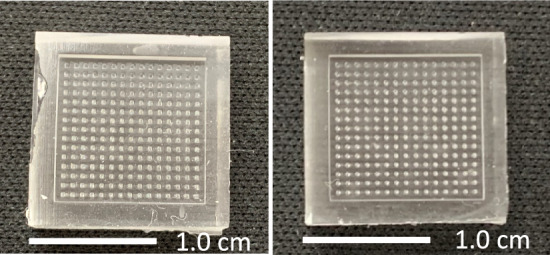
The gross appearance of prepared PDMS MN molds.

After PDMS MNs molds fabrication, all twelve 3D-printed MNs patches with four different shapes with distinct width base or diameter were successfully removed from PDMS molds without any breaking tip of needles left inside the mold cavities. The results illustrated that the prepared MNs molds were clear and transparent, featuring 15 × 15 needle holes as illustrated in Fig. 6.

### Physical characterizations of prepared MNs patches

#### Physical appearance

**Figure 7:**
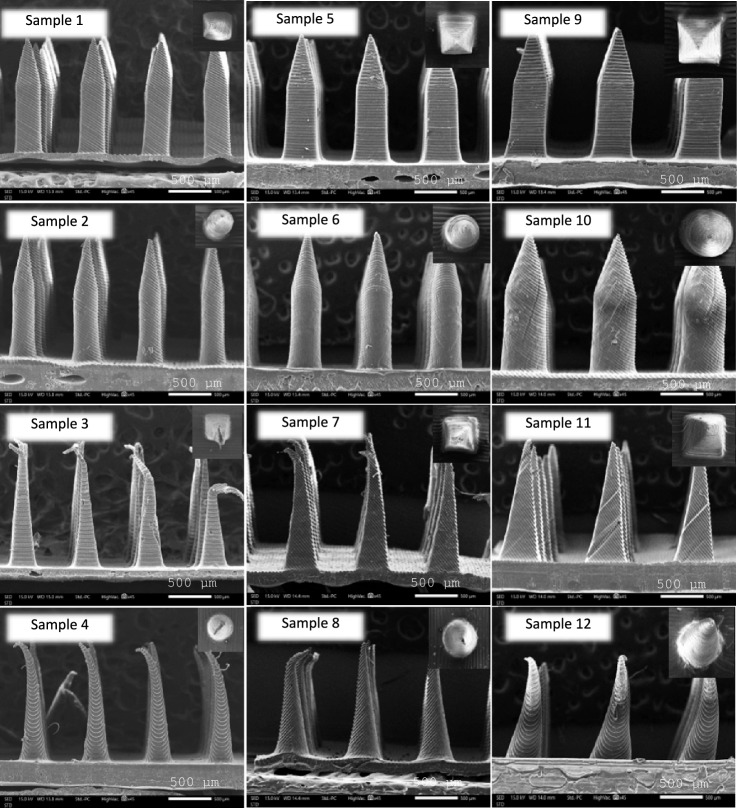
SEM images of twelve samples at 45 × magnification.

HPMC/PVP K90 DMNs structure was examined both top view and front view using an SEM at 45 × magnification to select the formulation with an acceptable physical appearance. Importantly, one of the essential parameters of DMNs is the ability to pierce through the skin because they must be able to penetrate stratum corneum for delivering drugs or substances. A bent or damaged needle indicates poor mechanical strength influencing skin insertion ability^31^. The results illustrated that sample 3, 4, 7, 8 and 12 had exactly bent tip while the others showed completed structure with sharp needles as shown in Fig. 7. Importantly, the needles of sample 3, 4, 7 and 8 collapsed after removing from PDMS mold. The decrease of width/diameter of the needle associates with the reduction of width/diameter tip which make the tip of the needle break or bend easily because it requires low force to destroy the needles. Moreover, the tip width/diameter is noted to be higher in MN that possesses larger width base/diameter, and those of higher tip width/diameter needs higher force to break the tip^30^. Both height and width/diameter of sample 1, 2, 5, 6, 9, 10 and 11 (Table 6) decreased when compared to those of 3D-printed MN patches because PDMS can shrink up to 2.5% after curing^32^ so the PDMS mold cavities decrease after separating 3D-printed MN patch and PDMS mold. During the drying process, the size of the needles decreased because of the loss of both DI water and ethanol^33^. Lastly, sample 1, 2, 5, 6, 9, 10 and 11 showed acceptable appearance so they were selected to perform % height reduction study.

**Table 6:** Base width/diameter (μm) and height (μm) of the selected samples.

Sample	Base width/diameter (μm)	Height (μm)	% height reduction	% blue dots
1 (shape A)	321.73 ± 10.81^a^	1298.03 ± 14.51^a^	16.25 ± 3.11^a^	–
2 (shape B)	325.30 ± 10.51^a^	1329.41 ± 11.30^b^	18.63 ± 3.87^a^	–
5 (shape A)	420.17 ± 11.63^b^	1403.55 ± 15.44^c^	12.22 ± 2.71^b^	–
6 (shape B)	415.76 ± 11.43^b^	1416.37 ± 18.75^c,d^	13.45 ± 1.93^b^	–
9 (shape A)	515.24 ± 15.54^c^	1447.79 ± 21.07^d^	7.46 ± 1.92^c^	99.41 ± 0.68^a^
10 (shape B)	517.66 ± 18.96^c^	1446.76 ± 23.71^d^	8.12 ± 2.42^c^	98.81 ± 1.28^a^
11 (shape C)	499.91 ± 15.06^c^	1387.83 ± 34.86^c^	18.24 ± 1.68^a^	–

For each test, the average values with the same letter in the same column show no statistical difference (*p* > 0.05).

#### Height reduction (%)

HPMC/PVP K90 DMNs strength was evaluated using texture analyzer to confirm that the needles can pierce the human skin without structural break. Moreover, the % height reduction should be less than 10% to exhibit great mechanical strength^3,34^. On the other word, mechanical strength was performed to eliminate the fragile formulation during the compression mode^31^. The results showed that the % high reduction of sample 1, 2, 5, 6 and 11 was 16.25, 18.63, 12.22, 13.45 and 18.24%, respectively as illustrated in Table 6, so these five samples indicated low mechanical strength, which may not be able to pierce the skin^3^ while only sample 9 and 10 had % high reduction less than 10%. However, to increase the mechanical strength of both shape C and D, the height of the designed MN patch using CAD Software must be reduced^31^. Interestingly, an increase in width or diameter resulted in a decrease % height reduction in both shape A and B. These results were related to the increase of tip diameter or width which correlated to the rise of base width or diameter of the needle^30^. Additionally, increasing the tip diameter or width also requires a higher amount of polymer solution to be used in printing higher layers, which increase the amount of monomer involved in the polymerization process, affecting the strength of the microneedle^15^. In conclusion, sample 9 and 10 had the lowest % height reduction, which were 7.46 and 8.12%, respectively with no statistically significant difference, so these two samples were selected to evaluate their skin insertion.

#### Skin insertion ability

**Figure 8:**
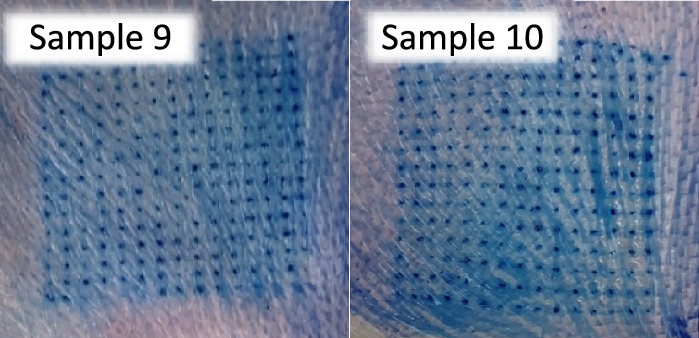
Blue dots appeared on neonatal porcine skin of sample 9 and 10.

The ability to insert the needles through the skin is one of the important factors in DMNs, and the characteristic of human skin, type of polymers, and the sharpness of the needles were the three major factors influencing insertion ability^35^. The presence of blue dots on neonatal porcine skin signified a successful insertion of the MNs through the skin^3^ as determined in Fig. 8. The results showed that sample 9 and 10 had their % blue dots of 99.41 and 98.81%, respectively with no significant difference (approximately 100%) as shown in Table 6. It can be concluded that low % height reduction resulted in high % blue dots which is correlated with Anjani et al.^34^ and Ramadon et al.^36^ results. In addition, pyramid mounted over a long cube (shape A) and cone mounted over a cylinder (shape B) DMNs patches showed better physical properties than pyramidal shape (shape C), and conical shape (shape D), so sample 9 and 10 can be used as a model to prepare DMNs incorporated drug.

## Conclusion

Stereolithography can be used to construct MNs patches with different shapes of needle designed from CAD Software. For square base MN, the optimal curing time and printing angle were 1.5 s and 30° whereas for round base, 2.0 s and 45° were the suitable curing time and printing angle. Additionally, all twelve designs of 3D-printed MN patches must print with anti-aliasing to obtain smooth needle edges. Moreover, those of 3D-printed MN patches were utilized to produce MN molds using PDMS, and twelve PDMS molds were used to manufacture HPMC/PVP K90 DMNs patches. Although HPMC/PVP K90 DMNs had smaller sizes compared to the designs using CAD software, they were able to pierce the skin. Moreover, DMNs patches that had the needle of shape A and B revealed better physicochemical properties than those of shape C and D. As a result, sample 9 and 10 can be used to prepare HPMC/PVP K90 DMNs incorporated drug. Future investigation of this study would be dissolving ability of each shape of DMNs, coupled with the corporation of drug in DMNs.

## Data Availability

The datasets used and/or analyzed during the current study are available from the corresponding author on reasonable request.
